# One-Year Myopia Control Efficacy of a New Defocus Spectacle Lens: A Randomized Clinical Trial

**DOI:** 10.1016/j.xops.2025.100940

**Published:** 2025-09-17

**Authors:** Xiaotong Han, Yurui Zhang, Ling Jin, Decai Wang, Mingguang He, Yangfa Zeng

**Affiliations:** 1 State Key Laboratory of Ophthalmology, Zhongshan Ophthalmic Center, Sun Yat-Sen University, Guangdong Provincial Key Laboratory of Ophthalmology and Visual Science, Guangzhou, Guangdong, China; 2 School of Optometry, The Hong Kong Polytechnic University, Kowloon, Hong Kong, China; 3 Research Centre for SHARP Vision (RCSV), The Hong Kong Polytechnic University, Kowloon, Hong Kong, China; 4 Centre for Eye and Vision Research (CEVR), Hong Kong, China

**Keywords:** Myopia control, Defocus spectacle lens, Refractive development, Axial elongation

## Abstract

**Purpose:**

To evaluate the efficacy and safety of the new defocus spectacle lens in preventing myopia progression compared with the conventional single-vision spectacle lens (SVL).

**Design:**

A randomized, open-label, controlled clinical trial.

**Subjects:**

Children aged 6 to 14 years with a cycloplegic spherical equivalent refraction (SER) of –1.00 to –3.50 diopters (D) in both eyes were enrolled.

**Methods:**

Eligible participants were randomly assigned in a 1:1 ratio to receive either the novel defocus spectacle lens (MYOGEN) or the SVL. Data from the right eyes at the 12-month follow-up were used for the current analysis.

**Main Outcome Measures:**

The primary outcome was the 1-year change in SER from baseline to 12 months. Secondary outcomes included changes in axial length (AL), choroidal thickness, subjective visual quality scores, and daily spectacle wear time. Axial length was designated as the main secondary outcome due to its close relationship with myopia progression.

**Results:**

A total of 85 patients were assigned to the MYOGEN group and 89 to the SVL group, with mean (standard deviation) ages of 10.44 (0.92) years and 10.61 (0.73) years, respectively. After 1 year, myopia progression and axial elongation were all significantly less in the MYOGEN group than in the SVL group (SER change: –0.80 ± 0.44 D and –1.06 ± 0.51 D, respectively; AL change: 0.25 ± 0.15 mm and 0.37 ± 0.18 mm, respectively; all *P* < 0.001). Among patients with good compliance (≥8 hours/day and ≥5 days/week), the treatment effect was even more pronounced for SER (adjusted difference: 0.40 D [95% confidence interval [CI], 0.25 to 0.56]) and AL (adjusted difference: –0.15 mm [95% CI, –0.21 to –0.10]). No significant between-group differences were observed in compliance with spectacle wear and subjective visual quality.

**Conclusions:**

Compared with the SVL, MYOGEN was shown to reduce myopia progression and axial elongation over the 1-year follow-up period.

**Financial Disclosure(s):**

Proprietary or commercial disclosure may be found in the Footnotes and Disclosures at the end of this article.

The prevalence of myopia has been increasing globally.^1^^,^^2^ By 2050, the global population with myopia is projected to reach 4758 million (49.8% of the world population), with high myopia affecting approximately 938 million individuals (9.8% of the global population).^2^ Uncorrected myopia has become a leading cause of visual impairment, and high myopia is associated with an elevated risk of irreversible visual impairment such as glaucoma and retinal detachment,^3^^,^^4^ contributing to the overall disease burden and adversely affecting quality of life.^5^^,^^6^ Therefore, slowing the progression of myopia is considered a public health priority.

Previous studies have demonstrated that pharmacological,^7^, ^8^, ^9^, ^10^ environmental,^11^ and optical interventions^12^, ^13^, ^14^, ^15^ can effectively control myopia. The efficacy of atropine is dose-dependent, though higher concentrations are associated with adverse effects such as photophobia and glare due to cycloplegia.^7^^,^^10^ A randomized controlled trial in children from Guangzhou indicated that increased outdoor time reduced the risk of myopia onset,^11^ although its effect on myopia progression remains controversial.^16^^,^^17^ Among current optical strategies, orthokeratology, defocus soft contact lenses, and defocus spectacle lenses are commonly used. However, the infection risk associated with orthokeratology and contact lenses limits its widespread application.^18^^,^^19^ The theoretical foundation for optical interventions lies in evidence from animal models, where peripheral myopic defocus has been shown to inhibit axial elongation.^20^, ^21^, ^22^, ^23^ Based on this mechanism, various defocus lenses have been developed and implemented in clinical practice.

Lam et al reported that Defocus Incorporated Multiple Segments (DIMS) spectacle lenses, designed with multiple small defocus zones, were able to slow myopia progression by approximately 50% over a 2-year period.^15^ This effect is attributed to the induction of full-field peripheral myopic defocus, which helps counteract hyperopic defocus in all directions. Previous studies have indicated that DIMS lenses have minimal or no effect on various aspects of visual function, such as distance and near visual acuity, accommodative response, stereopsis, and horizontal phoria.^15^^,^^24^^,^^25^ However, because the visual image is segmented by multiple small defocus zones, DIMS lenses may induce visual disturbances, such as image vibration and light scatter, which could compromise visual clarity and comfort.^26^ And studies have shown that children wearing DIMS lenses experienced symptoms such as peripheral blur, headaches, and halos during the adaptation period.^27^

Recently, a novel defocus spectacle lens (MYOGEN), similar in principle to the DIMS lens, has been developed to suppress hyperopic defocus across all directions. The aim of this study was to compare the 1-year efficacy of this novel defocus spectacle lens with that of conventional single-vision spectacle lens (SVL) in controlling myopia progression among children.

## Methods

### Study Design

This 12-month, 2-arm, open-label randomized controlled trial was conducted in Guangzhou, China, from March 23, 2023, to April 22, 2024, with participants recruited from local primary schools. Written consent was obtained from participants' parents or legal guardians prior to enrollment. The study protocol was approved by the Institutional Review Board of Zhongshan Ophthalmic Center, Guangzhou, China (identifier, 2023KYPJ006), and was registered at clinicaltrial.gov (identifier, NCT05740904). All procedures adhered to the tenets of the Declaration of Helsinki. The study was designed and reported in accordance with the Consolidated Standards of Reporting Trials guidelines.

Participants who met the inclusion and exclusion criteria were recruited for the study. Inclusion criteria were as follows: (1) age between 6 and 14 years; (2) cycloplegic spherical equivalent refraction (SER) between –1.00 diopters (D) and –3.50 D with astigmatism ≥ –1.50 D in both eyes; (3) absolute interocular difference in SER ≤1.00 D; (4) binocular best-corrected visual acuity ≥1.0, as determined by subjective refraction; and (5) intraocular pressure between 10 and 21 mmHg in both eyes. Exclusion criteria included (1) presence of ocular diseases such as cataract, glaucoma, fundus pathology, ocular trauma, manifest strabismus, or any other condition affecting visual function; (2) systemic diseases associated with immunosuppression or psychiatric disorders that may impair cooperation; and (3) a history of myopia control interventions (e.g., orthokeratology and atropine) or participation in any myopia control clinical trial within the past 3 months.

Eligible participants were enrolled and randomly assigned in a 1:1 ratio to either the MYOGEN group (intervention) or the SVL group (control) using a stratified block randomization method. Stratification was based on school and grade level, and allocation was performed using a computer-generated randomization list by an independent statistician. Due to the visible differences between the 2 types of spectacles, the trial was conducted in an open-label manner. The study investigator and optometrist were masked to the group allocation. Study visits were conducted at baseline, as well as at the 6-month and 12-month follow-up.

### Interventions

The control group received SVLs, while the intervention group received MYOGEN spectacle lenses (developed and manufactured by TOKAI OPTICAL CO, LTD). All participants were instructed to wear their assigned spectacles for ≥5 days per week and a minimum of 12 hours per day.

**Figure 1:**
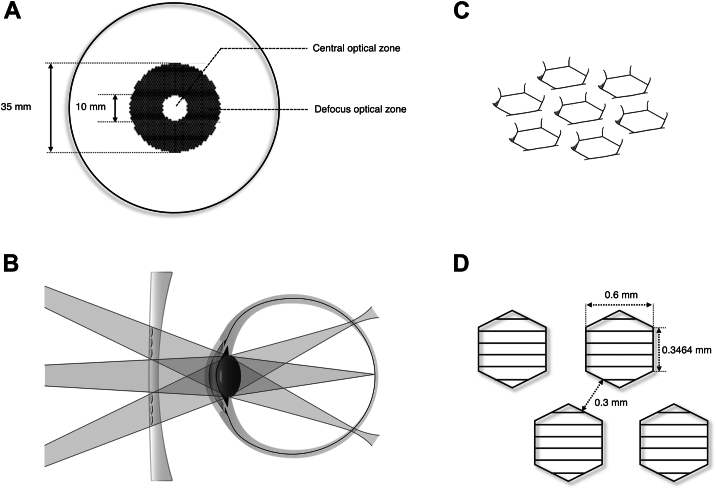
Optical design of the MYOGEN spectacle lens. **A,** Lens structure comprising a central optical zone and a surrounding defocus optical zone. **B,** Diagram illustrating the light path through the lens onto the retina. **C,** Perspective view of the microlens array. **D,** Plan view of the microlens array.

The MYOGEN spectacles in this study were specifically engineered to induce full-field peripheral myopic defocus, thereby suppressing hyperopic defocus caused by off-axis aberrations in all directions. The lens features a central optical zone of approximately 10 mm dedicated to refractive correction, surrounded by a defocus zone extending up to 35 mm in diameter. Within the defocus zone, a convex band with a roughly cylindrical structure is formed by approximately 1330 surrounding hexagons. The distance between opposite sides of the hexagon is approximately 600 μm, and the width of the cylindrical structure is approximately 300 μm. The change from the 2 straight focus lines in the front and back directions of astigmatism to the point image distribution of precise focus is very gentle, so there will be no sharp point image change like the DIMS lens, which can inhibit the discomfort of the field of vision being divided. The induced myopic defocus reaches a positive cylindrical power of ≥ +5.00 D in the regions between the edges of adjacent hexagons (Fig 1). This optical configuration is designed to effectively control axial elongation by increasing the depth of focus while avoiding significant degradation of peripheral retinal image quality.^28^^,^^29^

In both groups, participants were eligible for a free replacement of their spectacles if any of the following conditions were met at each follow-up:

1. A change in SER of ≥0.50 D in either eye or a decrease of ≥2 lines in corrected visual acuity compared with the previous visit.
2. Spectacle damage or any other condition judged by the investigator to require spectacle replacement.

### Measurements and Outcomes

At baseline, in addition to recording participants' demographic characteristics, visual acuity testing and slit lamp examinations were performed. Intraocular pressure was assessed using a noncontact tonometer (Canon TX-10). Uncorrected visual acuity was measured prior to cycloplegia by a certified optometrist using the ETDRS logarithm of the minimum angle of resolution E chart (Precision Vision). For participants with uncorrected visual acuity <1.0, best-corrected visual acuity was additionally measured. After slit lamp examination, eligible participants received 2 drops of 1% cyclopentolate hydrochloride in each eye, applied 5 minutes apart. Cycloplegia was considered complete if the light reflex was absent and the pupil diameter was ≥6 mm; otherwise, a third drop was administered. Cycloplegic SER was measured using an autorefractor (Topcon KR8800), and the final value was calculated as the average of 3 consecutive measurements.

The primary outcome of this study was cycloplegic SER, assessed at baseline and the 12-month follow-up. Secondary outcomes included ocular biometry, choroidal thickness (ChT), subjective visual quality score, and daily duration of spectacle wear, which were measured at baseline and 6-month and 12-month follow-up visits. Among these, axial length (AL) was the main secondary outcome due to its direct association with myopia progression. Ocular biometry, including AL, anterior chamber depth, corneal curvature, and lens thickness, was measured prior to cycloplegia using a noncontact optical biometer (IOLMaster 700; Carl Zeiss Meditec). Each parameter was measured 5 times per eye, and the mean value was used for analysis. Choroidal thickness was assessed using OCT (Zeiss Cirrus 5000), with the average of 12 measurements used for analysis. The subjective visual quality score was obtained using the Pediatric Refractive Error Profile 2 questionnaire.^30^ The daily duration of spectacle wear was recorded at each follow-up visit based on reports from children or their guardians. Adverse events and serious adverse events were monitored and recorded at each follow-up visit.

### Sample Size

To achieve 85% power to detect a 0.50 D difference in SER progression between the 2 groups, assuming a standard deviation of 1.00 D and a 2-sided alpha level of 0.05, the minimum required sample size was 73 participants per group. Assuming an anticipated dropout rate of approximately 15%, a total of 86 subjects were required in each group, resulting in 172 participants overall. Sample size calculation was performed using PASS software, version 16.0 (NCSS, LLC).

### Statistical Analysis

Data from the right eyes were used for the current analysis. Analyses will be conducted based on the full analysis set (FAS) using the modified intention-to-treat (mITT) principle, as well as on the per-protocol set for per-protocol analysis. The FAS excluded participants who did not initiate spectacle wear after randomization or were lost to both the 6-month and 12-month follow-up visits. The per-protocol analysis, derived from the FAS, further excluded participants who wore spectacles for <6 months or missed any follow-up visit. For the mITT analysis, a subgroup of participants with good compliance (wearing spectacles for ≥8 hours per day and ≥5 days per week) was analyzed to assess the treatment effect among those with optimal adherence. All baseline demographic characteristics were analyzed using the FAS, and safety evaluations were performed based on the safety analysis set.

Baseline characteristics and outcomes were compared between the intervention and control groups. For normally distributed continuous variables, independent t-tests were used. When normality assumptions were not met, the Mann–Whitney U test was applied. Categorical variables were analyzed using the χ^2^ test.

In addition to assessing between-group differences and reporting 2-sided 95% confidence intervals (CIs), changes in SER, AL, and ChT were further analyzed using linear regression models adjusted for baseline characteristics, including age, sex, baseline cycloplegic SER, and parental myopia status. Adjusted 95% CI was also reported. No adjusted analyses were conducted for the other secondary outcomes. The SER was defined as the spherical power plus half the cylindrical power. Myopia was defined as a cycloplegic SER of ≤ –0.50 D in either eye.

Exploratory subgroup analyses were performed for the primary outcome and axial elongation, with subgroups based on spectacle-wearing duration (≥8 vs. <8 hours/day; ≥12 vs. <12 hours/day) and weekly wearing frequency (≥5 vs. <5 days/week). This resulted in 6 subgroups: ≥8 hours/day, <8 hours/day, ≥12 hours/day, <12 hours/day, ≥8 hours/day with ≥5 days/week, and ≥12 hours/day with ≥5 days/week.

All statistical analyses were performed using STATA 16.0 (StataCorp).

## Results

**Figure 2:**
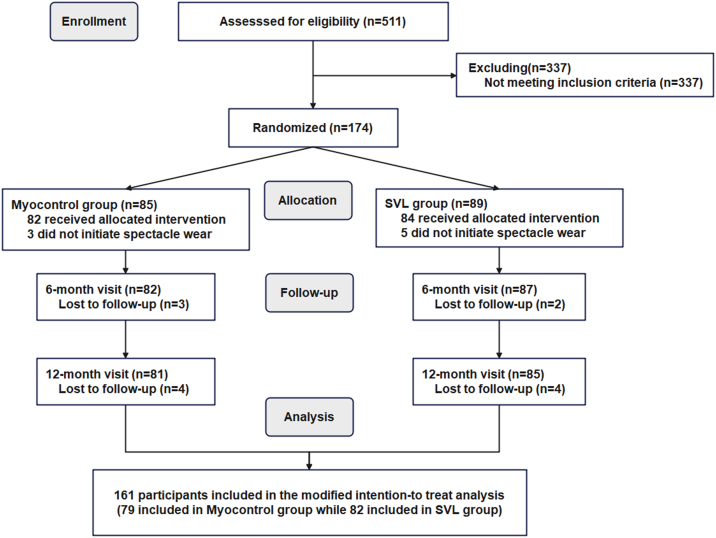
Flow diagram of the study design. SVL = single-vision spectacle lens.

Figure 2 presents a flow diagram outlining the number of participants recruited, enrolled, and excluded. A total of 174 eligible schoolchildren were enrolled and randomly assigned to the MYOGEN group (n = 85) or the SVL group (n = 89). In the intervention and control groups, 3 and 2 participants, respectively, missed both the 6-month and 12-month follow-up visits; 3 and 5 participants did not initiate spectacle wear after randomization; 1 and 2 participants missed only the 12-month follow-up; and 2 and 1 participants wore the spectacles for <6 months.

As a result, 161 participants were included in the mITT analysis (79 in the MYOGEN group and 82 in the SVL group), and 155 participants were included in the per protocol (PP) analysis (76 in the MYOGEN group and 79 in the SVL group). Among these, 57 participants in the MYOGEN group and 66 in the SVL group were classified as having good compliance and were analyzed separately in the mITT analysis to assess the treatment effect among those with optimal adherence.

No significant difference in baseline characteristics was identified between the 2 groups (Table 1). At baseline, the mean (standard deviation) SER was –2.15 (0.67) D in the MYOGEN group and –2.12 (0.76) D in the SVL group. The mean (standard deviation) baseline AL was 24.44 (0.82) mm and 24.40 (0.81) mm, respectively. The mean (standard deviation) baseline ChT was 261.75 (49.64) μm in the MYOGEN group and 269.83 (49.09) μm in the SVL group.

**Table 1:** Distributions of Baseline Characteristics (N = 161)

	MYOGEN (n = 79, 48.85%)	SVL (n = 82, 51.15%)
Age, yrs	10.44 (0.92)	10.61 (0.73)
Male sex	43 (54.43)	40 (48.78)
Number of parents with myopia		
0	32 (40.51)	36 (43.90)
1	16 (20.25)	17 (20.73)
Both	31 (39.24)	29 (35.37)
Height, cm	138.96 (9.51)	139.69 (8.25)
Weight, kg	36.01 (11.34)	36.51 (10.40)
Cycloplegic SER, D	–2.15 (0.67)	–2.12 (0.76)
Axial length, mm	24.44 (0.82)	24.40 (0.81)
Choroidal thickness, μm	261.75 (49.64)	269.83 (49.09)
Anterior chamber depth, mm	3.78 (0.23)	3.80 (0.19)
Lens thickness, mm	3.30 (0.14)	3.33 (0.17)
Corneal curvature, D	43.60 (1.51)	43.58 (1.66)
Axial length/corneal curvature ratio	3.15 (0.07)	3.15 (0.07)

D = diopters; SER = spherical equivalent refraction; SVL = single-vision spectacle lens.

Continuous data are presented as mean (standard deviation), and categorical data as counts (percentages).

### Changes in SER and AL

Based on the mITT analysis, over the 1-year follow-up period, the mean myopia progression, measured as the change in SER, was significantly lower in the MYOGEN group (–0.80 ± 0.44 D) compared with the SVL group (–1.06 ± 0.51 D). Similarly, the mean axial elongation was significantly less in the MYOGEN group (0.25 ± 0.15 mm) compared with the SVL group (0.37 ± 0.18 mm) (Table 2).

**Table 2:** Changes in SER and AL at 1 Yr Based on mITT Analysis

All Participants	MYOGEN (n = 79)	SVL (n = 82)	Unadjusted Between-Group Difference (95% CI)	Adjusted Between-Group Difference (95% CI)∗
Change in SER, D	–0.80 (0.44)	–1.06 (0.51)	0.26 (0.12, 0.41)†	0.29 (0.14, 0.44)†
Change in AL, mm	0.25 (0.15)	0.37 (0.18)	–0.11 (–0.17, –0.06)†	–0.13 (–0.18, –0.08)†

Good Compliance	MYOGEN (n = 57)	SVL (n = 66)	Unadjusted Between-Group Difference (95% CI)	Adjusted Between-Group Difference (95% CI)∗
Change in SER, D	–0.76 (0.41)	–1.11 (0.48)	0.35 (0.19, 0.51)†	0.40 (0.25, 0.56)†
Change in AL, mm	0.25 (0.15)	0.38 (0.17)	–0.13 (–0.19, –0.08)†	–0.15 (–0.21, –0.10)†

AL = axial length; CI = confidence interval; D = diopters; mITT = modified intention-to-treat; SER = spherical equivalent refraction; SVL = single-vision spectacle lens.

Data are presented as mean (standard deviation).

Good compliance = wearing spectacles for ≥8 hrs per day and ≥5 days per week.

∗ Adjusted for baseline characteristics, including age, sex, baseline cycloplegic SER, and parental myopia status.

† *P* < 0.001.

Among participants with good compliance, the MYOGEN group showed an even greater reduction in myopia progression, with a mean change in SER of –0.76 ± 0.41 D compared with –1.11 ± 0.48 D in the SVL group. Axial elongation was also significantly less in the MYOGEN group (0.25 ± 0.15 mm) compared with the SVL group (0.38 ± 0.17 mm).

The findings in participants with good compliance were even more pronounced than in the overall mITT analysis. After adjusting for baseline characteristics, including age, sex, baseline cycloplegic SER, and parental myopia status, the adjusted mean between-group difference in myopia progression was 0.40 D (95% CI: 0.25, 0.56; *P* < 0.001) for SER and –0.15 mm (95% CI: –0.21, –0.10; *P* < 0.001) for axial elongation. This corresponds to a 36.0% reduction in myopia progression and a 39.5% reduction in axial elongation, respectively, compared with the SVL group.

The findings were consistent in the per-protocol analysis, which excluded participants who wore spectacles for <6 months. In the PP analysis, the between-group difference in SER progression remained statistically significant after adjustment (adjusted mean difference: 0.29 D; 95% CI: 0.14, 0.44; *P* < 0.001) (Table S1, available at www.ophthalmologyscience.org).

### Other Secondary Outcomes

Based on the mITT analysis, the reduction in ChT was less pronounced in the MYOGEN group (–13.33 ± 24.07 μm) than in the SVL group (–25.43 ± 22.28 μm), with an unadjusted between-group difference of 12.09 μm (95% CI: 4.81, 19.38; *P* = 0.001). The MYOGEN group exhibited 47.58% less choroidal thinning compared with the control group. These results remained statistically significant after adjustment for baseline covariates (all *P*<0.01) (Table S2, available at www.ophthalmologyscience.org). And in the PP analysis, the between-group difference in ChT change remained statistically significant after adjustment (all *P* < 0.01) (Table S1).

There was no significant difference between the MYOGEN group and SVL group in the change of anterior chamber depth, lens thickness, or corneal curvature over the 12 months, whether based on mITT or the PP analysis. A significantly smaller increase in AL/corneal radius ratio was observed in the MYOGEN group compared with the SVL group in both mITT and PP analyses (between-group difference: –0.01; 95% CI: –0.02 to –0.006; *P* < 0.001) (Tables S3 and S4, available at www.ophthalmologyscience.org).

### Subgroup Analysis

Subgroup analyses further evaluated the association between spectacle wear time and treatment efficacy. In participants who wore spectacles for ≥8 or ≥12 hours per day, the MYOGEN group demonstrated significantly less SER progression compared with the SVL group (all *P* < 0.001). After adjusting for baseline characteristics, the between-group differences remained significant in both the ≥8 hours/day (0.38 D, 95% CI: 0.23, 0.53) and ≥12 hours/day (0.35 D, 95% CI: 0.19, 0.51) subgroups (*P* < 0.001). Likewise, participants who wore spectacles for ≥8 or ≥12 hours/day for ≥5 days/week exhibited significant between-group differences (*P* < 0.001). In contrast, no significant between-group differences were observed in subgroups with <8 or <12 hours/day of wear, regardless of baseline adjustment (Table 3).

**Table 3:** Changes in Cycloplegic SER after 12 Mos of Spectacle Wear in Participants with Different Wearing Durations Based on mITT Analysis

	MYOGEN	SVL	Unadjusted Between-Group Difference (95% CI)	Adjusted Between-Group Difference (95% CI)∗
≥8 hrs/day	–0.77 (0.41)	–1.10 (0.48)	0.33 (0.18, 0.49)†	0.38 (0.23, 0.53)†
<8 hrs/day	–0.82 (0.44)	–0.80 (0.61)	–0.02 (–0.43, 0.39)	–0.13 (–0.62, 0.36)
≥12 hrs/day	–0.76 (0.41)	–1.07 (0.46)	0.31 (0.14, 0.48)†	0.35 (0.19, 0.51)†
<12 hrs/day	–0.82 (0.42)	–1.02 (0.60)	0.20 (–0.10, 0.50)	0.16 (–0.16, 0.49)
≥8 hrs/day with ≥5 days/wk	–0.76 (0.41)	–1.11 (0.48)	0.35 (0.19, 0.51)†	0.40 (0.25, 0.56)†
≥12hrs/day with ≥5 days/wk	–0.74 (0.41)	–1.08 (0.46)	0.33 (0.16, 0.50)†	0.38 (0.21, 0.54)†

CI = confidence interval; mITT = modified intention-to-treat; SER = spherical equivalent refraction; SVL = single-vision spectacle lens.

Data are presented as mean (standard deviation). Changes in SER are expressed in diopters (D).

∗ Adjusted for baseline characteristics, including age, sex, baseline cycloplegic SER, and parental myopia status.

† *P* < 0.001.

### Compliance, Subjective Visual Quality Assessment, and Adverse Events

Over the past year, 78.95% of participants in the intervention group wore spectacles for ≥8 hours per day, and 68.42% for ≥12 hours per day. In the control group, the corresponding proportions were 84.81% and 67.09%, respectively. Additionally, 81.58% of participants in the intervention group and 91.14% in the control group wore spectacles on ≥5 days per week (Table S4). There was no significant difference in compliance between the 2 groups (*P* > 0.05).

Regarding subjective visual quality, no statistically significant difference was observed between the intervention and control groups (58.34 ± 11.53 vs. 59.54 ± 11.42, *P* = 0.525).

Adverse events were reported in 5 participants (6.33%) in the intervention group and 4 participants (4.88%) in the control group. The primary adverse events included abnormal intraocular pressure and vertical or horizontal misalignment between the optical center or fitting reference point and the pupil reflex point. Spectacle-related adverse events occurred in 2 participants in each group (MYOGEN: 2.53%; SVL: 2.44%). No adverse events led to study withdrawal or were classified as serious in either group.

## Discussion

This study showed that MYOGEN spectacle lenses significantly slowed myopia progression and axial elongation in schoolchildren compared with SVLs. Among participants with good compliance (wearing spectacles for ≥8 hours per day and ≥5 days per week), the MYOGEN group exhibited 36.04% less myopia progression and 39.47% less axial elongation. The findings were consistent across both mITT and PP analyses and remained robust after adjustment for key baseline covariates. No serious adverse events were reported.

Several studies have evaluated the efficacy of defocus lenses in controlling myopia progression. One study involving 193 children in Hong Kong reported the 1-year SER progression of –0.17 D (relative to SVL: 69.09% reduction) with DIMS lenses incorporating a +3.50 D relative positive power.^15^ A clinical practice–based study study conducted at Aier Eye Hospital found the 1-year SER progression of –0.50 D (relative to SVL: 35.06% reduction) in children wearing the same type of lenses.^31^ Another study investigating novel spectacle lenses with highly aspherical lenslets reported the 1-year SER progression of –0.30 D (relative to SVL: 63.29% reduction).^32^ Compared with the 3 previous studies, our findings indicate a greater rate of myopia progression in both the intervention and control groups. This difference may be explained by variations in baseline SER, compliance, and participant recruitment sources. In our study, participants had a less myopic baseline SER (MYOGEN vs. SVL: –2.15 D vs. –2.12 D) compared with previous studies, including the Hong Kong DIMS study (–2.93 D vs. –2.70 D), the Aier Eye Hospital study (–2.93 D vs. –2.67 D), and the highly aspherical lenslets lens study (–2.70 D vs. –2.46 D). As suggested in a meta-analysis, greater baseline myopia was associated with enhanced treatment efficacy of defocus spectacle lenses.^33^ Moreover, while prior studies recruited participants from clinical settings, our study enrolled children from primary schools. Compared with participants recruited from clinical settings, those enrolled from schools are more representative with lower spectacle-wearing compliance, which directly affects treatment efficacy. The spectacle-wearing compliance in the highly aspherical lenslets study was higher (daily wear >12 hours) than in the current study, whereas compliance was not documented in the other 2 studies.

We found that, in addition to slowing SER progression and axial elongation, MYOGEN spectacle lenses also significantly reduced choroidal thinning. Previous studies have shown that children with myopia progression exhibit marked decreases in ChT.^34^ The choroid may play a role in modulating scleral growth by regulating ocular blood perfusion, thereby influencing myopia progression.^35^ By mitigating choroidal thinning, these lenses may help preserve ocular structural integrity.

In subgroups with spectacle wear of ≥8 or ≥12 hours per day and on ≥5 days per week, SER progression was significantly lower in the MYOGEN group than in the SVL group. The similar degrees of myopia progression observed across the 4 subgroups suggest that wearing the lenses for ≥5 days per week and ≥8 hours per day is sufficient to achieve a significant myopia control effect. These findings indicate that wearing MYOGEN spectacle lenses during regular school hours alone may provide an effective intervention for schoolchildren.

Although the MYOGEN spectacle lenses incorporated a relative positive power of +5.00 D, subjective visual quality did not differ significantly between the MYOGEN and SVL groups. This may be attributed to the novel optical design of the lens, which minimizes visual discomfort associated with a split or disrupted visual field.

There were some limitations in our study. First, it was conducted exclusively in Chinese children, with participants limited to an SER range of –1.00 to –3.50 D, thereby neglecting potential ethnic differences and the effects of MYOGEN spectacle lenses in children with high myopia. Second, daily spectacle wear time was self-reported and may be subject to recall bias; future studies could incorporate wearable devices to enhance measurement accuracy. Finally, the follow-up duration was relatively short, and the long-term benefits of MYOGEN spectacle lenses warrant further investigation.

## Conclusions

The MYOGEN spectacle lenses significantly reduced myopia progression and axial elongation in schoolchildren compared with SVLs. The lenses preserved good visual quality and were well tolerated.
