# Supplementary material for: One-Year Myopia Control Efficacy of a New Defocus Spectacle Lens: A Randomized Clinical Trial
Source: Ophthalmol Sci. 2025 Sep 17;6(1):100940. doi: 10.1016/j.xops.2025.100940 (PMC12607012; doi:10.1016/j.xops.2025.100940)
Supplement: Tables S1–S4 [file mmc1.pdf]

Supplementary materials

Supplementary table 1. Changes in SER, AL and ChT at 1 Year Based on Per-Protocol Analysis

	MYOGEN (n = 76)	SVL (n = 79)	Unadjusted between-group difference (95% CI)	Adjusted between-group difference (95% CI) †
Change in SER, D	-0.80 (0.44)	-1.07 (0.51)	0.26 (0.11, 0.41)***	0.29 (0.14, 0.44)***
Change in AL, mm	0.25 (0.15)	0.37 (0.18)	-0.12 (-0.17, -0.06)***	-0.13 (-0.18, -0.08)***
Change in ChT, μm	-13.18 (24.31)	-25.43 (22.42)	12.25 (4.83, 19.66)**	11.94 (4.40, 19.49)**

Data are presented as mean (standard deviation).

† Adjusted for baseline characteristics, including age, sex, baseline cycloplegic SER, and parental myopia status.

CI = confidence interval; D = diopters; SVL = single-vision lens;

SER = spherical equivalent refraction; AL = axial length; ChT = choroidal thickness;

*** = P < 0.001; ** = P < 0.01; * = P < 0.05.

Supplementary table 2. Changes in ChT at 1 Year Based on mITT Analysis

All Participants	MYOGEN (n = 79)	SVL (n = 82)	Unadjusted between-group difference (95% CI)	Adjusted between-group difference (95% CI) †
Change in ChT, μm	-13.33 (24.07)	-25.43 (22.28)	12.09 (4.81, 19.38)**	11.74 (4.34, 19.14)**

Data are presented as mean (standard deviation).

† Adjusted for baseline characteristics, including age, sex, baseline cycloplegic SER, and parental myopia status.

mITT = modified intention-to-treat; CI = confidence interval; SVL = single-vision lens;

*** = P < 0.001; ** = P < 0.01; * = P < 0.05.

Supplementary table 3. Changes in ACD, LT and corneal curvature at 1 Year Based on mITT Analysis

	MYOGEN (n = 79)	SVL (n = 82)	Unadjusted between-group difference (95% CI)	P-value
Change in ACD, mm	0.01 (0.04)	0.01 (0.11)	-0.001 (-0.03, 0.02)	0.924
Change in LT, mm	0.02 (0.02)	0.02 (0.02)	0.001 (-0.006, 0.008)	0.787
Change in corneal curvature, mm	0.01 (0.13)	-0.02 (0.14)	0.03 (-0.009, 0.08)	0.119
Change in AL/CR	0.03 (0.02)	0.05 (0.02)	-0.01 (-0.02, -0.006)	< 0.001

Data are presented as mean (standard deviation).

mITT = modified intention-to-treat;

SD = standard deviation; CI = confidence interval; SVL = single-vision lens;

ACD = anterior chamber depth; LT = lens thickness; AL/CR = Axial length/corneal curvature ratio.

Supplementary table 4. Changes in ACD, LT and Corneal Curvature at 1 Year Based on Per-Protocol Analysis

	MYOGEN (n = 76)	SVL (n = 79)	Unadjusted between-group difference (95% CI)	P-value
Change in ACD, mm	0.01 (0.04)	0.03 (0.04)	-0.01 (-0.03, -0.001)	0.031
Change in LT, mm	0.02 (0.02)	0.02 (0.02)	0.0005 (-0.007, 0.008)	0.898
Change in corneal curvature, mm	0.01 (0.13)	-0.008 (0.12)	0.02 (-0.02, 0.06)	0.270
Change in AL/CR	0.03 (0.02)	0.05 (0.02)	-0.01 (-0.02, -0.006)	< 0.001

Data are presented as mean (standard deviation).
mITT = modified intention-to-treat;
SD = standard deviation; CI = confidence interval; SVL = single-vision lens;
ACD = anterior chamber depth; LT = lens thickness; AL/CR = Axial length/corneal curvature ratio.

Supplementary table 5. Spectacle Wear Compliance Based on Per-Protocol Analysis

	Myocontrol, n (%) (n = 76)	SVL, n (%) (n = 79)
Weekly Spectacle Wearing Time		
< 8 hours/day	16 (21.05)	12 (15.19)
8-12 hours/day	8 (10.53)	14 (17.72)
≥ 12 hours/day	52 (68.42)	53 (67.09)
Weekly Spectacle Wearing Time		
< 5 days/week	14 (18.42)	7 (8.86)
≥ 5 days/week	62 (81.58)	72 (91.14)

Data are presented as counts (percentages).
SVL = single-vision lens.
